# Assessment of pediatric eye care services in health facilities in the Ashanti region of Ghana

**DOI:** 10.4102/phcfm.v17i1.4972

**Published:** 2025-09-16

**Authors:** Elizabeth M. Akpakli, Alvin J. Munsamy, Nishanee Rampersad

**Affiliations:** 1 Discipline of Optometry, School of Health Sciences, University of KwaZulu-Natal, Durban, South Africa

**Keywords:** visual impairment, provision, primary eye care services, children, health facilities

## Abstract

**Background:**

Childhood visual impairment is an important public health concern considering the social, emotional and economic consequences. Lack of access to eye care services contributes to this growing problem. Therefore, integrating primary eye care into existing primary healthcare would facilitate improved access to equitable, effective and affordable eye care services, particularly for children.

**Aim:**

The study assessed the provision of primary paediatric eye care services in health facilities in the Ashanti region of Ghana.

**Setting:**

The study was conducted at the primary health facilities in the Ashanti region of Ghana.

**Methods:**

Stratified random sampling was used to select 145 health facilities in this descriptive study. The eye care professionals in these facilities completed a questionnaire concerning primary eye care services for children. Data were analysed using descriptive and inferential statistics.

**Results:**

Eye care services were available in 131 (90%) of the health facilities resourced with essential eye equipment. Refraction services were provided by 129 (98.5%) despite limited coverage by the national health insurance scheme. More than 80% of participants lacked continuing education and 59% had poor awareness of management guideline. Barriers identified included lack of specialised equipment and inadequate resources.

**Conclusion:**

The study highlights disparities in the provision of child eye care services among the health facilities in the Ashanti region of Ghana.

**Contribution:**

This study provides useful information to inform policy on targeted interventions for child eye care services to ensure accessible, equitable and comprehensive services.

## Introduction

The World Health Organization (WHO) reports that approximately 19 million children are visually impaired and 1.4 million are blind.^1^ Children with visual impairment experience social, emotional and economic challenges and are less likely to attend school and attain employment.^2^ Childhood visual impairment is a critical consideration not only for the number of affected children but also because of the number of years the surviving child must live with the visual impairment.^3^ Lack of access to eye care services is recognised as an important reason for why people remain or become blind.^4^ The prevalence of vision impairment in low- and middle-income regions is four times higher than in high-income regions.^5^ Therefore, there is a need for low- and middle-income countries (LMICs) to provide universal access to eye care, not just for sight threatening conditions but also for conditions that cause functional limitations. Researches globally support the integration of primary eye care into existing primary healthcare (PHC) systems as a viable solution to improving access and reducing avoidable blindness.^6,7^ In this regard, PHC forms an integral part of the health system in a country and a central point for social and economic development of every community.^8^ Primary healthcare is designed to be the first level of health service contact for individuals, families and communities and the national health system at large.^9^ In this model, appropriately trained health personnel can diagnose and manage common eye conditions at the community level and refer more complex cases through an established care pathway.^10^ The WHO’s Global Action Plan for Universal Eye Health (2013–2019) highlights the role of PHC workers in delivering primary eye care and emphasises the need for capacity-building and systems integration. Despite this, literature specific to Ghana remains limited, particularly regarding the availability of paediatric eye care services at the PHC level.

Therefore, the aim of this study was to assess the provision of primary paediatric eye care services in health facilities in the Ashanti region of Ghana. These results will provide valuable insights into the current lack of data and help guide primary eye care planning and policy making for child eye care.

## Research methods and design

### Study design

The study used a cross-sectional design to assess the provision of eye care services among children in primary health facilities in the Ashanti region of Ghana. The health facilities were visited once, and different cadres of eye care professionals, such as ophthalmologists, optometrists, ophthalmic nurses and opticians, were assisted to complete a semi-structured questionnaire.

### Study area and population

The study was conducted in the Ashanti region of Ghana located in the southern part of Ghana. The region has 43 districts and 195 sub-districts. The Ashanti region of Ghana has a well-developed health sector with 530 health facilities comprising Community-Based Health Planning and Services (CHPS) facilities, health centres, clinics, maternity homes, district hospitals and teaching hospitals. Most of these health facilities are operated by the Ghana Health Service (*n* = 170) and private institutions (*n* = 281) with the remaining by missions (*n* = 71) and the Ashanti quasi-government (*n* = 8). The population comprised eye care professionals from the primary health facilities.

### Sample size and sampling technique

The health facilities were selected using stratified random sampling, where health facilities in each district were considered as the strata and selected for inclusion by simple random sampling. Stratified sampling with equal allocation was applied to select the health facilities from each district. Four health facilities were randomly chosen from each district, resulting in a total of 172 health facilities. At the health facilities, an eye care professional, such as an optometrist, ophthalmic nurse and optician, was then selected randomly to fill the questionnaire. Health facilities that did not provide eye care services were excluded – facilities that do not provide services designed to prevent, diagnose and treat paediatric eye conditions.^11^ The eye care professionals were eligible for the study if they were working at the facility for at least 3 years prior to the period of data collection.

### Data collection

Data collection was over a period of 17 months from March 2023 to July 2024. The questionnaire used for data collection was developed based on validated scales from previous research.^12^ It was semi-structured, consisting of both open-ended and close-ended questions. The questionnaire was piloted on 16 participants, not included in the final sample, where input for improvement was given. The questionnaire (Appendix A) was modified based on feedback received concerning question format, type, order and flow to suit the study objective. After providing consent, the eye care professionals were assisted, via teaching and research assistants, to fill the paper questionnaire. Data from the completed paper questionnaire were thereafter entered into a Google Form. Data were extracted from the paper questionnaire for the following broad themes, including demographic data, knowledge and skills about child eye services, type of equipment available and cost of eye care services and if these services were covered by the National Health Insurance Scheme (NHIS).

### Data analysis

Quantitative data were extracted into Microsoft Excel and analysed with STATA 14. Descriptive statistics, including means, standard deviations (s.d.), percentages and frequency distributions, are used to summarise demographic information and survey responses. The chi-square test was used to test the association between years of professional practice and awareness of guideline. A *p*-value of less than 0.05 was considered statistically significant.

### Ethical considerations

Ethical clearance to conduct this study was obtained from the Kwame Nkrumah University of Science and Technology, Kumasi Committee on Human Research, Publication and Ethics (No. CHRPE/AP/771/22) and the University of KwaZulu-Natal Humanities and Social Sciences Research Ethics Committee (HSSREC) (No. HSSREC/00004574/2022). Gatekeeper permission was also sought from the regional health directorate and the heads of the selected health facilities. Participation was voluntary, and all participants provided informed consent before the study began.

## Results

### Demographic characteristics

A total of 145 health facilities were assessed where one eye care professional working in each facility was contacted to fill the questionnaire, resulting in 145 eye care professionals. This comprised 95 (65.5%) males and 50 (34.5%) females. The mean age (s.d.) of the participants was 32.5 (6.13) years. The eye care professionals included 101 (69.7%) optometrists, 26 (17.9%) ophthalmic nurses, 17 (11.7%) opticians and 1 (0.7%) ophthalmologist. More than 60% of participants (*n* = 91) had less than 10 years of practice experience (Table 1).

**TABLE 1:** Demographic characteristics of participants.

Variable	Frequency (*n*)	Percentage (%)
**Gender**		
Male	95	65.50
Female	50	34.50
**Age (years)**		
< 25	7	4.76
25–29	44	29.93
30–34	36	24.49
35–39	50	34.01
> 40	8	6.80
**Profession**		
Ophthalmologist	1	0.70
Optometrist	101	69.70
Ophthalmic nurse	26	17.90
Optician	17	11.70
**Years of practice (years) (*n* = 142)**		
< 5	74	52.10
5–9	17	12.00
10–15	46	32.40
> 15	5	3.50

### Range and availability of child eye care services

Table 2 shows the range of services available for children in the health facilities. The majority of facilities provided refraction services (*n* = 129, 98.5%), followed by binocular vision services (*n* = 68, 51.9%), low vision services (*n* = 43, 32.8%), 15 (11.5%) other services such as colour vision test and seven (5.3%) provided surgical services. In instances where eye care services were not available, complex eye conditions were referred to (*n* = 23), while basic eye conditions were managed (*n* = 6) at their facilities. The majority of participants reported organising school screenings (*n* = 113).

**TABLE 2:** Range of child eye care services.

Variable	Frequency (*n*)	Percentage (%)
**Type of child eye care services (*n* = 131)**		
Refraction services	129	98.5
Binocular vision services	68	51.9
Low vision services	43	32.8
Other services	15	11.5
Surgical services	7	5.3
**School screening (*n* = 138)**		
Yes	113	81.9
No	25	18.1
**Compensatory action for no child eye service (*n* = 29)**		
Manage basic eye conditions	6	20.7
Refer complex conditions	23	79.3

**FIGURE 1:**
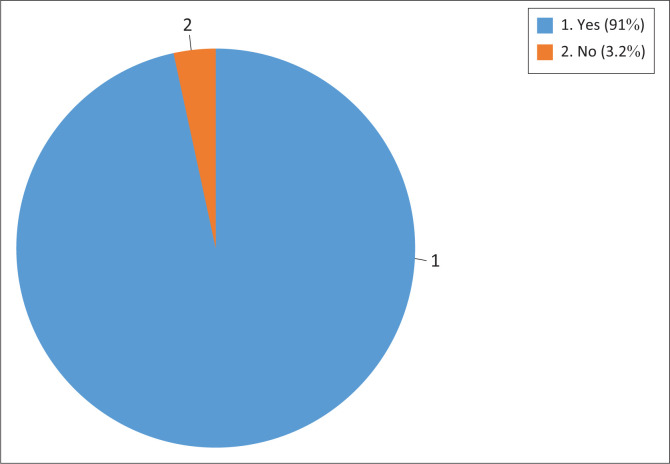
Availability of child eye care services.

Figure 1 shows the availability of child eye care services in their health facilities as reported by participants (*n* = 144) and range of eye care services. Of these, more than 90% provided eye care services for children (*n* = 131), while the remaining (*n* = 13) did not provide any services for children.

### Resources for child eye services

**FIGURE 2:**
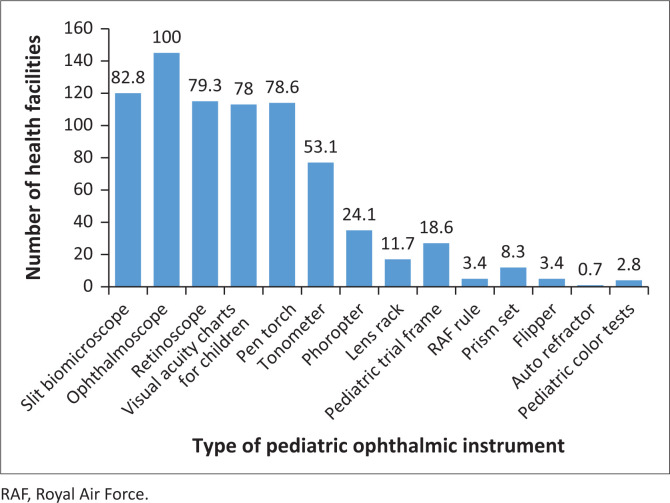
Equipment available for child eye care services at facilities (*n* = 143).

Figure 2 presents the availability of equipment necessary for the provision of eye care services for children in the different health facilities. All participants reported that an ophthalmoscope was available. As shown in Figure 2, a high proportion of the facilities also had slit lamp biomicroscopes (82.8%), retinoscopes (79.3%), pen torches (78.6%), visual acuity charts for children (78%) and tonometers (53.1%), while a lower proportion of facilities had phoropters (24.1%), paediatric trial frames (18.6%), lens rack (11.7%), prism set (8.3%), Royal Air Force (RAF) rules (3.4%), flippers (3.4%), paediatric colour charts (2.8%) and autorefractors (0.7%).

### Knowledge and skills about child eye services

As shown in Table 3, 22 (15.2%) participants indicated that they had additional training in child eye care services, of only nine were for management of child conditions, six for low vision and rehabilitation services and three for paediatric dispensing. Participants (84.8%) who lacked additional training stated different reasons for this, including lack of finances (10.42%), lack of interest (15.6%), lack of opportunity (53.12%), few years in practice and paediatrics not included in job description (20.8%).

**TABLE 3:** Knowledge and additional training of participants.

Variable	Frequency (*n*)	Percentage (%)
**Additional training (*n* = 145)**		
Yes	22	15.20
No	123	84.80
**Area of additional training (*n* = 18)**		
Low vision and rehabilitation in children	6	33.30
Paediatric dispensing	3	16.70
Management of paediatric eye cases	9	50.00
**Reasons for no additional training (*n* = 96)**		
Financial barrier	10	10.42
Lack of interest	15	107.30
Lack of opportunity	51	53.12
Others	20	20.83
**Awareness of routine guideline (*n* = 137)**		
Yes	56	41.00
No	81	59.00
**Type of guideline (*n* = 42)**		
Visual acuity	7	17.00
Routine comprehensive eye examination	13	31.00
AOA guideline for children	18	43.00
Guideline on refraction and dispensing	4	9.00
**Continuous professional education in child eye care (*n* = 143)**		
Yes	27	18.90
No	116	81.10

AOA, American Optometric Association.

Approximately 40% of participants were aware of guidelines for the management of eye conditions in children (Table 3). When asked about the type of guideline, the American Optometric Association (AOA) guideline was most commonly reported (*n* = 18), followed by a routine comprehensive eye examination guideline (*n* = 13), visual acuity guideline (*n* = 7) and refraction and dispensing guideline for children (*n* = 4). Less than 20% of participants engaged with continuous professional educational activities related to child eye services (Table 3).

There was no significant association found between the two groups by experience greater or less than 10 years and awareness of guideline ($\chi_{\left(3,137\right)}^{2}$ = 0.751, *p* = 0.4).

### Sponsorship of child eye services

**FIGURE 3:**
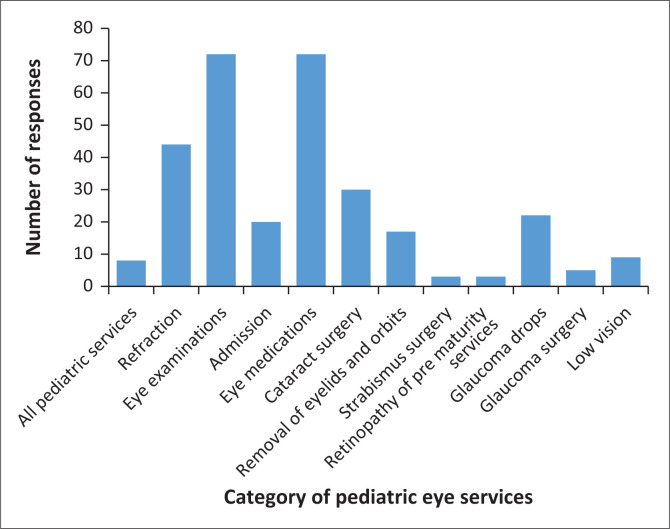
Category of eye care services in children sponsored by National Health Insurance Scheme.

The majority of participants noted that the NHIS does not sponsor child eye care services (*n* = 107, 74.8%). For those who indicated NHIS coverage, eye examinations and eye medication were most commonly mentioned, while other services and fees including refraction, cataract surgery and payment for admission were also mentioned, as shown in Figure 3.

### Challenges and barriers to eye care services in children

Table 4 shows that the barriers to the provision of eye care services for children included specialised equipment as the main barrier while inadequate public education, specialised human resources and cost were lesser barriers. Barriers to the uptake of child eye care services included unavailable human and physical resources as the major barriers to uptake, while delays in seeking treatment by parents and guardians, cost of services and poor accessibility to eye clinics because of distance were considered as minor barriers.

**TABLE 4:** Challenges and barriers to the provision of eye care services in children.

Variable	Frequency (*n*)	Percentage (%)
**Barriers**		
Specialised equipment	36	54.00
Cost	3	4.50
Specialised human resources	11	16.00
Inadequate education	14	21.00
Others	3	4.50
**Gaps in the uptake of child eye services**		
Cost	13	16.00
Unwilling parents or guardians	17	21.00
Poor accessibility	7	8.64
Unavailable resources	32	39.50
Others	12	14.80

## Discussion

The study sought to report on the provision of primary eye care services among children in health facilities in the Ashanti region of Ghana. Overall, eye services for children were available in the region, with refraction services being the most common and provided mostly by optometrists working in these health facilities. Despite this, most eye care professionals indicated not having additional training and managed basic conditions while referring complex conditions for further management. In this study, most of the eye care professionals were males aligning with workforce trends reported in a similar study in Southern Ethiopia.^13^ There were more optometrists and ophthalmic nurses in the sample suggesting that these eye care professionals served as the providers of primary eye care services in the communities. A recent study^14^ noted a similar trend and stated that optometrists and ophthalmic nurses are vital role players in providing primary eye care services. Furthermore, the eye care workforce in the Ashanti region of Ghana is relatively young, considering that most participants had less than 10 years of professional experience.

### Range and availability of child eye care services

Early detection and intervention for eye conditions among children are crucial for improving global child health outcomes. In this study, there was a high level of provision of child eye care services, as more than 90% of participants reported providing these services. This finding is encouraging and aligns with efforts aimed at prioritisation of avoidable childhood blindness considering the profound impact of visual impairment in children.^12^ However, the absence of child eye care services in some of the health facilities suggests an inequity of services in some communities underscoring the need for the ministry of health to intervene and implement interventions to ensure that eye care services are available and accessible in all health facilities. A structured referral system was used in health facilities that lacked child eye care services where complex eye conditions were referred, ensuring that affected children received advanced care. This approach has also been used previously where community nurses identify people with vision impairment and advise on non-optical interventions, make referrals and facilitate access to advanced low vision services.^15^ It demonstrates the importance of referral systems to bridge service delivery gaps that may exist to ensure that the appropriate services are assessed and afforded.

Majority of participants reported organising school screening activities that are an essential and effective strategy for the early detection and management of eye conditions in school children. Such strategies aim to reduce the impact of visual problems and improve academic performance in children, particularly in LMICs.^16^ Almost all health facilities provided refraction services indicating that detection and correction of refractive error is widely available. This can be attributed to the high number of optometrists in the districts unlike in a study in Zambia where refractive error correction services were limited.^17^ The provision of eye care services for refractive error is crucial, as uncorrected refractive error (URE) is a readily treatable cause of visual impairment globally.^18^ Binocular vision services were the second most common type of child eye care services and support a previous study that highlighted the availability of such services in Ghana.^19^ The provision of binocular vision services helps detect and effectively manage ocular problems, indirectly improving academic performance among children. Low vision services, such as assessments and provision of assistive devices, were available at approximately one-third of the health facilities, which is concerning given the increasing prevalence of vision impairment worldwide. This gap in the provision of low vision services is well known, as it is one of the most underprovided services globally, especially in Africa.^15^ Early identification and treatment of eye conditions among children are crucial for their visual and cognitive development; hence, it is recommended that policymakers prioritise provision of low vision services by improving funding for equipment and staff training. The focus on refraction services, while critical, suggests an imbalance in service provision. Therefore, it is recommended that eye care professionals undergo additional training and that health facilities be better equipped to offer comprehensive primary eye care services for children.

### Resources for child eye services

The role of human and physical resources is crucial for delivering accessible and high-quality eye care services. In general, most health facilities had basic diagnostic equipment that are necessary to provide eye care services. Ophthalmoscopes, which enable examining the ocular health, were available at all health facilities as has been reported previously.^20^ This availability and the use of ophthalmoscope are essential for accurate diagnosis and management of various posterior ocular conditions. A high proportion of health facilities were also equipped with slit lamp biomicroscopes and retinoscopes, which are also critical for ocular health and refraction procedures, respectively. A recent study^21^ in the Pacific also noted a similar finding among mid-level eye care workers concerning accessibility of slit lamp biomicroscopes and retinoscopes. Additionally, other tools including pen torches and visual acuity charts specific for children were widely available similar to findings in a study in Oman.^22^ Taken together, the availability of these equipment supports the examination and diagnostic processes involved in child eye care services. Fewer facilities had tonometers indicating a potential gap in assessment of intraocular pressure in children that is important for diagnosing glaucoma. The inadequate availability of tonometers could delay diagnosis of paediatric glaucoma, increasing the likelihood of irreversible optic nerve damage and visual impairment. This study noted limited availability of advanced tools, such as phoropters, paediatric trial frame, lens racks, prism sets, RAF rules, flippers, paediatric colour charts and autorefractors, which are needed to effectively assess and manage a wide range of visual functions and refractive errors in children. This lack of additional equipment for providing comprehensive eye care services for children creates potential barriers to the availability of good-quality primary eye care services. Overall, the disparity regarding equipment in some of the health facilities underscores the need for improved resource distribution and increased funding to enhance eye care service delivery and achieve universal eye coverage in the Ashanti region of Ghana.

### Knowledge and skills of eye care professionals about child eye services

Engaging in competency-based and continuous education is crucial to improve patient care and provide sustainable high-quality eye care. Less than 20% of participants had additional training in child eye care services, highlighting a significant gap in specialised paediatric eye care and provision of its services. Similar results have been found in studies involving primary eye care providers in another African country.^13^ The lack of additional training for child eye care may lead to inefficient services, and staff should be encouraged to engage in courses, workshops and webinars focused on paediatric eye care to expand and update their knowledge and skills. The major reasons noted for lack of additional training were lack of opportunity and lack of interest. Lack of opportunity may be because of limited access to training programmes and resources. Without adequate resources, even motivated professionals may struggle to receive the necessary education and hands-on experience. Preference for other specialties and perceived low priority may cause lack of interest in child eye care. This aligns with a previous study^23^ that noted child eye care not being a priority in many African healthcare systems. Hence, it is essential to expand child eye care training programmes and integrate them into existing health education systems. Funding for infrastructure and scholarships should be also increased to improve access to specialised training. Additionally, raising awareness about the importance of child eye care through continuing professional development initiatives may help generate greater interest in this field.

One in every four participants was aware of guidelines for managing eye conditions in children. The varying guidelines followed in these facilities may lead to inconsistent diagnostic and treatment outcomes for eye conditions among children. To address the adoption and use of varying guidelines, it is recommended that all eye care providers in these health facilities undergo training using a standardised evidence-based curriculum for child eye care services. Furthermore, mechanisms should be implemented, following the training, to monitor adherence to the principles and guidelines contained in the curriculum. It is concerning to note that less than 20% of participants engaged in continuing professional education activities related to child eye services, as such activities help eye care professionals remain updated to provide evidence-based care. In Tanzania, PHC staff displayed improved knowledge in eye care after undergoing periodic training programmes.^24^ In this study, there was no association between years of experience and awareness of management guidelines for child eye conditions. In contrast, a study suggests that awareness and familiarity with eye examination guidelines improves with age and experience.^25^

### Sponsorship of child eye services

Health insurance schemes are recognised as a useful tool to finance provision of healthcare services in developing countries with the potential to increase utilisation of these services, better protect against health expenses and address issues of equity.^26^ Most of the participants reported that the NHIS does not sponsor child eye care services reflecting a knowledge gap among these eye care professionals as eye care services are covered by the NHIS in Ghana.^26^ Hence, there is a need to improve awareness about NHIS policies and benefits concerning eye care services among eye care professionals and patients. Additionally, regular audits of NHIS implementation should be conducted in health facilities to assess compliance and identify inconsistencies. Eye examinations and medications were noted as common benefits of the NHIS consistent with a previous finding that the NHIS in Ghana covers some eye care services, but not spectacles and optical devices.^27^ The limited coverage for refractive and cataract surgery services is concerning because such services are necessary to curb the common treatable causes of visual impairment, particularly among children in LMICs. Therefore, there is a need for better engagement of policymakers on the public health and economic benefits of covering refractive and cataract surgery services in Ghana.

### Challenges and barriers to child eye services

Barriers to accessing child eye care services children are wide ranging and encompass factors that hinder children from obtaining these services leading to negative consequences.^28^ The lack of specialised equipment, high cost of services, limited specialised human resources and inadequate public education were noted as barriers to the provision of child eye services in this study. Inadequate specialised equipment and limited human resources significantly affect the provision of child eye care services in developing countries as has been noted in a study in Swaziland.^29^ To overcome this, the ministry of health, private sectors and non-governmental organisations should collaborate and work more efficiently towards providing essential ophthalmic instruments and staff in the health facilities to enhance service provision. High cost of services was noted as a barrier to accessing service, and this was also noted in the present study. To address this, NHIS should expand its coverage to make eye care services more affordable for poor families, and private sponsors should be contacted to organise free community eye screening programmes to help identify eye problems for prompt interventions. Similarly, gaps concerning the uptake of these services included cost of services, unwillingness of parents to seek prompt interventions, poor accessibility because of long distances to eye clinics and unavailable human and physical resources. Delayed presentation because of parental attitude and distance can cause further deterioration and potentially lead to visual impairment in children. Consequently, community education programmes to raise and improve awareness about the importance of early detection and prompt intervention for eye conditions are necessary. Additionally, it is recommended that community outreach activities be organised in underserved populations to improve access to eye care services in an effort to reduce the burden of avoidable visual impairment.

### Limitations

The results of this study reflect the provision of child eye care services in health facilities from only one region of Ghana and therefore cannot be generalised to the whole country. Additionally, the exclusion of facilities that do not provide eye care services could have introduced a selection bias consequently preventing generalisation of the study findings. Furthermore, this study used a survey with data being self-reported and could have introduced recall bias. Additionally, this cross-sectional study does not provide an in-depth understanding of the participants’ responses. Therefore, future studies should use qualitative methods of data collection such as interviews and focus groups to investigate provision of primary eye care services among children in these health facilities.

## Conclusion

Child eye care services were available in the Ashanti region of Ghana, with refractive services being the most common with notable gaps being in low vision provision. Disparities need to be addressed to ensure that equitable and comprehensive child eye care services are available to reduce the prevalence of avoidable visual impairment beyond refractive error. Few participants had further training and knowledge of paediatric eye care guidelines suggesting that targeted interventions are needed to improve knowledge, skills and quality of child eye care services. The limited NHIS coverage for child eye care services presents a significant barrier, and expanding the scope of NHIS and improving awareness of covered services are critical towards achieving equitable and sustainable child eye care services. Understanding and addressing the barriers to the provision and uptake of child eye care services are essential for improving accessibility and equity, which can potentially reduce the burden of avoidable visual impairment in children.

## Appendix A: Questionnaire for eye care professionals

You are being invited to consider participating a research that aims to assess the provision of pediatric eye care services in health facilities in the Ashanti Region of Ghana.

The findings will help improve the quality of eye care provided for children in the Ashanti region of Ghana.

No risks are anticipated which involves the completion of a 10-15 minutes semi-structured questionnaire. Your time for participating is greatly appreciated. Your confidentiality is fully assured as no information related to your identity will be taken. You are under no obligation to participate and can withdraw anytime during the study, even after you have agreed to participate.

1.0 Do you agree to participate in this study?
  Yes
  No
2.0 **Demographic Data**
  This section seeks to obtain information on your demographic characteristics. Select and provide short answers where necessary.
2.1 What is your age (years)?
2.2 What is your gender?
  Male
  Female
2.3 What is your profession?
  - Ophthalmologist
  - Optometrist
  - Ophthalmic Nurse
  - Optician
2.4 How many years have you practiced as an eye care professional?
2.5 Have you had any further training in pediatric eye care?
  Yes
  No
2.6 If yes, what training/qualification did you have? Write NA if No.
2.7 If yes, how many years after your first degree did you acquire this training?
2.8 How many years have you been practicing with this specialist training?
2.9 If you answered No to question 2.5, what possible reason could explain your lack of further training in pediatric eye care.
3.0 **Pediatric Eye Care Services**
  This section seeks to obtain information on the provision of pediatric eye care services in your facility
3.1 Does your facility provide pediatric eye care services. If No, continue from question 4.1
  Yes
  No
3.2 If yes, what pediatric eye care services are provided? Check all that apply
  - Low vision
  - Binocular vision
  - Refraction
  - Others, state
3.3 Who provides these services in your facility? Tick all that apply
  - Low vision specialist
  - Pediatric ophthalmologist
  - Pediatric Optometrist
  - Binocular vision Specialist
  - Optometrist
  - Ophthalmologist
  - Ophthalmic nurse
  - Specialist eye nurse
  - Optician
  - Others, state
3.4 Are you aware of any guideline for routine eye examinations for children?
  Yes
  No
3.5 If yes, what guideline do you follow?
3.6 If you answered No to question 3.1, why are pediatric services not offered in your facility?
3.7 If your facility does not provide pediatric eye care services, what do you do when a pediatric patient present to your facility?
3.8 Indicate any recognized needs in the provision or uptake of pediatric eye care services in your facility. Give details
3.9 Do you think the services provided for children are affordable?
3.10 Does your facility organize school eye screening in your district?
  Yes
  No
3.11 What is the average number of children that presents with eye problems to your facility daily?
3.12 Do you provide refractive services for children?
  Yes
  No
3.13 What is the typical waiting time it takes to see an eye care professional for spectacle prescription for children?
3.14 What is the average cost for basic good-quality prescription glasses inclusive frame and lenses?
3.15 Indicate any gaps in the provision or uptake of pediatric eye care services.
4.0 **Equipment**
  This section seeks to provide information on the equipment and materials used to provide pediatric eye care services
4.1 Tick the equipment that are available in your facility
  - Visual acuity charts for children
  - Retinoscope
  - Ophthalmoscope
  - Phoropter
  - Pen torch
  - Tonometer
  - Slit Lamp Biomicroscope
  - Others, please state
5.0 **Affordability**
  This section seeks to know whether pediatric eye care services are sponsored by government.
5.1 Are eye services for children sponsored by the government?
  Yes
  No
5.2 Select the categories of services that are mostly covered by the government?
  - All services including the ones below are provided by government establishment
  - Refraction
  - Provision of spectacles in a selected price range and type
  - Comprehensive eye examination
  - Admitted to stay at an eye inpatient ward after major/minor surgery
  - Eye medication
  - Low vision services
  - Glaucoma surgery
  - Cataract Surgery
  - Removal of eyelid growth and cyst
  - Strabismus surgery
  - Retinopathy of prematurity
  - Others, please state
6.0 **Training**
  This section seeks to obtain information on your tertiary training and continuing professional education.
6.1 Do you think the training you had form your tertiary institution had equipped you enough to handle pediatric eye cases in your clinic daily?
  Yes
  No
6.2 Is there a compulsory continuing professional education program in your facility for eye care professionals in pediatric eye case management?
  Yes
  No
